# Histopathological analysis of the non - tumour parenchyma following radical nephrectomy: can it predict renal functional outcome?

**DOI:** 10.1590/S1677-5538.IBJU.2016.0417

**Published:** 2017

**Authors:** Rana Birendra, Nirmal Thampi John, Neelaveni Duhli, Antony Devasia, Nitin Kekre, Ramani Manojkumar

**Affiliations:** 1 Department of Urology, Christian Medical College, Vellore;; 2 Department of Pathology, Christian Medical College, Vellore

**Keywords:** Nephrectomy, Arteriosclerosis, Glomerulosclerosis, Focal Segmental

## Abstract

**Introduction:**

Radical nephrectomy (RN), a recommended treatment option for patients with Renal cell carcinoma (RCC) leads to an inevitable decline in global renal function. Pathological changes in the non-tumour parenchyma of the kidney may help predict the function of the remaining kidney.

**Materials and Methods:**

Aim of this prospective, observational study was to find histopathological factors in the non-tumor renal parenchyma that could predict the decline in global renal function postoperatively and its association with co-morbidities like diabetes (DM). Data of consecutive patients undergoing RN from December-2013 to January-2015 was collected. Non-tumor parenchyma of the specimen was reported by a dedicated histopathologist. eGFR was calculated using Cockcroft-Gault formula before the surgery and at last follow up of at least 12 months.

**Results:**

73 RN specimens were analyzed. Mean follow up was 12.3 months. The mean decrease in eGFR was 22% (p=.0001). Percent decrease in eGFR did not show association with any of the histopathological parameters studied. DM was significantly associated with decrease in percent eGFR (p<0.05) and increase in arteriolar hyalinosis (p=0.004), Glomerulosclerosis (p=0.03) and Interstitial fibrosis/ Tubular atrophy (p=.0001). Maximum size of the tumor showed a negative correlation with percentage change in eGFR (p=.028).

**Conclusion:**

Histological parameters in the non-tumour portion of the RN specimen may not be able to predict renal function outcome over a short follow up. However, presence of DM was associated with adverse pathological changes and significant decrease in renal function postoperatively.

## INTRODUCTION

Radical nephrectomy (RN) is one of the recommended treatment options for patients with renal cell carcinoma (RCC). However, loss of a functioning kidney leads to adaptive hyperfiltration in the remaining kidney (1) resulting in glomerulosclerosis (2, 3). This insult to the kidney is further exacerbated by conditions like diabetes mellitus (DM) and hypertension (HTN). These changes are reflected in patient’s global renal function which shows a significant decline in 13% to 36% of patients (4). The pathological changes in the non-tumour parenchyma of the kidney may be helpful in predicting the clinical outcome in terms of renal function of the remaining kidney. An early detection of these factors and its timely management can delay the onset of kidney damage and the consequent patient morbidity. The aim of this study was to prospectively look at histological parameters in the non-tumour parenchyma of RN specimens and correlate it with the change in the patient’s eGFR over a period of at least 6 months. We also looked at the effect of DM and HTN on the non-tumour parenchyma and change in global renal function postoperatively.

## MATERIALS AND METHODS

**Figure 1:**
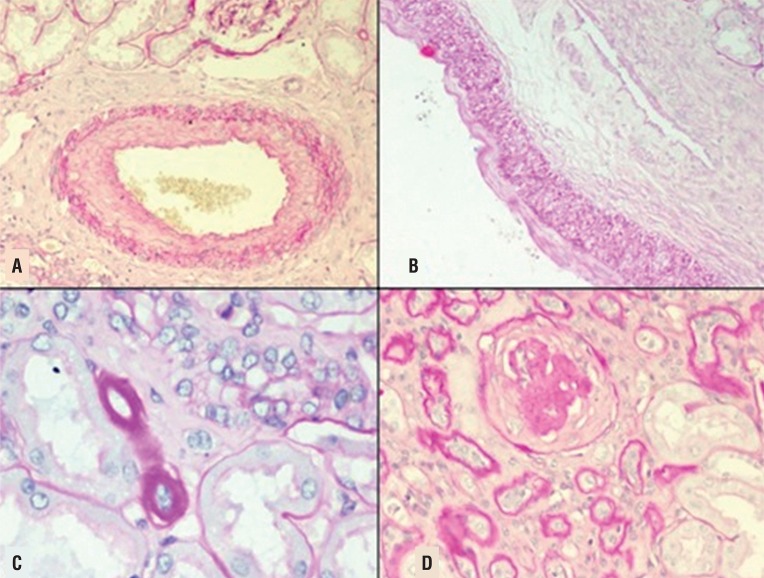
Histopathological factors (H&E): 1A) Arteriosclerosis- Intima more than media; 1B) Arteriosclerosis: Intima less than media; 1C) Arteriolar hyalinosis and 1D) Globally sclerosed glomeruli with tubular atrophy.

This prospective, observational study was carried out in the departments of Urology and Pathology, from Dec 2013 till Jan 2015. Institutional review board and ethics committee approval was obtained. All patients >18 years of age undergoing RN were consented and enrolled. Their clinical data was collected preoperatively, within 1 month of surgery and at last follow-up. A nephrectomy specimen in which the whole kidney was replaced by tumour was excluded. Sections of the non-tumour renal parenchyma for evaluation were taken at least 2cm away from the tumour margin to avoid any effect of tumour on the tissue. The biopsy specimen apart from haematoxylin and eosin (H&E) was also stained with periodic-acid Schiff (PAS). The specimen was reported by a dedicated histopathologist who was blinded to the patient’s clinical details. The histological factors evaluated were vascular, glomerular and tubule-interstitial based on the Oxford classification for IgA nephropathy (5) (Figure-1).

Arteriosclerosis (AS) was scored based on most severe lesions involving the interlobular arteries. It was graded based on the intimal and medial thickening leading to narrowing of the vascular lumen: none, intima less than media and intima more than media. Arteriolar hyalinosis (AH) was noted as proportion of arterioles affected: none; mild- ≤25%; moderate- 26% to 50% and severe- >50%. For statistical analysis it was grouped into two groups as less than 25% and more. Glomerulosclerosis (GS) was graded as Diffuse: a lesion involving most (≥50%) glomeruli; Focal: a lesion involving <50% of glomeruli; Global: a lesion involving more than half of the glomerular tuft; Segmental: a lesion involving less than half of the glomerular tuft. Glomerulosclerosis and Interstitial fibrosis/Tubular atrophy (IF/TA) was evaluated as percentage estimated to the nearest of 5%. For statistical analysis GS was graded into less than 10% and more and IF/TA was graded into 5% and more. Preoperative calculation of eGFR was done by the Cockcroft-Gault formula: **[eGFR= {140-age (year)}×weight(kg)/{72×serum creatinine (mg/dl)}×0.85 if female].** Post operatively eGFR was calculated again at 6 months or at last follow-up whichever was later and percentage change in eGFR calculated.

Sample size: Gautam et al. (6) reported that the change in eGFR from the baseline was nearly 31%. The standard deviation was 28.4mL/min. An assumption was made that greater decline in eGFR occurred in patients with more glomerulosclerosis, and change in those with least glomerulosclerosis would be as low as 10% to 15%. Keeping alpha and beta error at 5% and 10% respectively; and the power of the study at 90, the sample size calculated was 73.

### Statistical analysis

Data was entered using EPIDATA software version 3.1 and analysed using SPSS version 16. For preoperative characteristics of patients, p values were determined by the Mann-Whitney test for continuous variables and by χ2 test for categorical variables. As we had different categories of histological types to study the differences we used ANOVA test. Multivariable regression analyses having percent change in eGFR as dependent variable was done to control the effect of baseline variables such as DM, HTN etc.

## RESULTS

Eighty-six RNs were carried out from December 2013 to January 2015 of which, 73 were available for analysis. The base line characteristics of the patients and tumours are summarised in Table-1.

**Table 1:** Baseline characteristics of patients.

Age (years)	55.4±10.7 *
**Sex (%)**	
Male	56 (76.7)
Female	17 (23.3)
**BMI**	24.36±3.5 *
**Follow up (months)**	12.3±2.6 *
**Comorbidities (%)**	
DM	23 (31.5)
HTN	44 (60.3)
DM+HTN	18 (24.6)
**Type of Surgery (%)**	
Open	25 (34.2)
Laparoscopic	48 (65.8)

*mean ± standard deviation

The mean age of patients was 55.4 years with majority being male. They were followed up for an average period of 12.3 months. Hypertension was the most common co-morbidity. The histopathological findings are summarised in Table-2.

**Table 2:** Histopathological findings.

Histopathological factors	n (%)
**Glomeruloscleosis (GS)**	
≤10%	68(93.2)
>10%	5(6.8)
**Arteriolar hylinosis (AH)**	
None	7(9.6)
≤25%	42(57.5)
>25%-≤50%	15(20.5)
>50%	9(12.3)
**Arteriosclerosis (AS)**	
Intima<Media	4(5.5)
Intima>Media	69(94.5)
**Interstitial fibrosis/ Tubular atrophy (IF/TA)**	
≤5%	32(43.8)
5-≤10%	19(26.0)
>10-≤50%	13(17.8)
>50%	9(12.3)

As expected, clear cell carcinoma was the most common pathology. Average size of tumour was 7.3cm. T1 tumours accounted for 50% of the RNs, of which majority were stage T1b.

The mean eGFR in preoperative period was 81.8±29.6mL/min and postoperative period was 63.5±20.9mL/min. The mean decrease in eGFR was 22% (p=0001) 16 (22%) patients in the preoperative period had eGFR less than 60mL/min compared to 36 (49.3%) postoperatively 21 (28.8%) patient’s eGFR fell below 60mL/min compared to eGFR in the preoperative period. The mean fall in this group was 28.25±9.97mL/min (p=015). On linear regression analysis the percentage change in eGFR was not significantly affected by AS (p=0.7), AH (p=0.8), GS (p=0.4) or IF/TA (p=0.5). However, linear regression analysis showed that the percentage change in eGFR was significant in those affected by DM (p=05). Patients who had both DM and HTN also showed a significant decrease in eGFR compared to those who had either one or none (p=0.04). Maximum size of the tumour (sotl) showed a negative correlation with percentage change in eGFR change (p=03). In other words, smaller the tumour size greater the change in percentage eGFR. Other parameters like sex (p=0.9), HTN alone (p=0.8), grade of tumour (p=0.3), pre-operative ESR (p=0.6) did not significantly affect the percentage eGFR change.

DM had a significant association with presence of AH (p=0.004), GS (p=0.03) and IF/TA (p=0001). Presence of both DM and HTN also had a highly significant impact on arteriolar hyalinosis (p=004), GS (p=0.03) and IF/TA (p=0.001). However, HTN alone had no significant effect on AS (p=0.4), GS (p=0.72) and IF/TA (p=0.90). This was so because most of the patients with diabetes also had hypertension.

On multivariate analysis, the only factor which significantly affected the percentage change in eGFR was DM (Table-3).

**Table 3:** Univariate and Multivariate analysis with Percentage Change in eGFR as constant.

Risk Variables	Univariate		Multivariate	

	β	p-value	β	p-value
Age	- 0.25	0.22	- 0.36	0.16
Sex	0.37	0.94	0.64	0.92
BMI	0.26	0.68	- 0.05	0.95
Size of tumor	- 1.92	0.03	- 2.66	0.03
Arteriolar hyalinosis	- 4.32	0.35	- 6.88	0.24
Glomerulosclerosis	5.10	0.55	0.02	0.99
IF/TA	- 2.59	0.59	- 1.38	0.82
Arteriosclerosis	3.11	0.74	4.01	0.69
Diabetes Mellitus	- 9.25	0.04	- 14.78	0.02
Hypertension	- 0.88	0.84	3.56	0.52
Type of surgery	4.01	0.38	- 6.49	0.35
Blood loss	- 0.003	0.76	-0.005	0.64

**BMI =** Body mass index; **IF/TA =** Interstitial fibrosis/ Tubular atrophy; **Size of tumour =** Size of tumour in maximum dimension, Type of surgery (lap vs. open).

## DISCUSSION

Pathological changes in the non-tumour parenchyma of the kidney may be helpful in predicting the clinical outcome in terms of renal function of the remaining kidney. The basis of this is the presumption that the changes in the non-tumour parenchyma of the nephrectomy specimen will be reflection of the parenchyma of the remaining kidney which is left with the patient (4). The studies till date which have tried to address this possibility are fraught with limitations such as small numbers (6) and retrospective design (4). We evaluated the non-tumour portion of the RN specimens based on standard protocol to report medical renal disease (5). The renal function was measured using CG formula, as according to Kim et al., the CG model based on actual weight was 1 of 5 models that accurately estimates renal function in patients with a kidney tumour (7). The idea was to accurately document the parameters so that they can be objectively measured. We looked at the AH, AS, GS and IF/TA and did not find any significant association with the percent eGFR change. Gautam et al. in a retrospective study have shown that GS extent was associated with decrease in eGFR over a mean follow-up of 19.7 months (6). But, our study did not find the same. This can be because our follow-up period was short (mean of 12.3 months). This also could be because we used a different system of reporting the non-tumour parenchyma histology (5). Also, the studies done by Gautam et al. did not mention the type of staining used for slides. We used PAS stain for slides as recommended by the College of American Pathologists for the reporting of the surgically resected specimens of renal cell carcinoma (8).

Huang et al. reported that 26% of the patients in their cohort had pre-existing chronic kidney disease (CKD), defined as eGFR <60mL/min, before nephrectomy (9) and 70% of patients developed new onset CKD after RN over a mean follow-up of 19 months. In our study, 28.8% [21] patient’s eGFR fell below 60mL/min compared to pre-nephrectomy values with a mean fall of 20.34±8.6mL/min over a mean follow-up of 12.3 months. Bijol et al. found that patients with severe histopathological findings like parenchymal scarring >20%, global glomerulosclerosis and advanced diffuse diabetic glomerulosclerosis showed a significant change in serum creatinine from the preoperative period to 6 months after the RN (10). In our study, we found that patients with DM showed a significant change in percent eGFR over a period of 6 months. Diabetic patients showed significant adverse renal parenchymal changes in terms of AH, GS and IF/TA compared to non-diabetics. This could explain the significant change in post nephrectomy renal function of diabetic patients although no straight association between parenchymal parameters and percentage eGFR was found. HTN failed to show any significant association with percent change in eGFR.

Size of the tumour in maximum dimension showed a significant negative correlation with percent change in eGFR. This could be explained by the fact that in kidneys with smaller tumours, the normal functioning renal parenchymal loss is higher when compared to large tumours where the normal functioning parenchyma is considerably replaced by tumour. Our findings support the fact that tumours of size up to 7cm (T1) if amenable must be dealt with nephron sparing surgery. And, the presence of DM should strongly tilt the decision towards nephron sparing surgery in such patients.

The renal parenchymal characteristics in our study did not appear to significantly impact functional outcomes at one year of follow-up. It may be due to the fact that many of the patients had normal kidney function pre-operatively or did not have significant parenchymal damage. Only 5 patients in our study cohort had significant GS and this could have had a bearing on the interpretation of results. We did not do immunofluorescence and electron microscopic examination, to which diagnostic kidney biopsies are routinely subjected. Hence, our pick-up rate of medical renal disease may have been lower. Nevertheless, reporting of the non-tumour portion of the renal parenchyma should preferably be carried out routinely as it would give us an insight regarding the “quality” of the remaining renal mass and its long-term behaviour. Incorporating other methods of testing renal quality such as looking at the presence of proteinuria, imaging characteristics etc. may improve the probability of predicting renal functional outcome more accurately.

## CONCLUSIONS

Global renal function preservation should be aimed for in all the patients undergoing renal ablation surgery. Histological parameters in the non-tumour portion of the RN specimen may not be able to predict renal function outcome over a short follow-up. However, presence of DM was associated with adverse pathological changes and significant decrease in renal function postoperatively.
